# Self-Fetal Wellbeing Monitoring and Ante-Natal Care during the COVID-19 Pandemic: A Qualitative Descriptive Study among Pregnant Women in Indonesia

**DOI:** 10.3390/ijerph182111672

**Published:** 2021-11-06

**Authors:** Restuning Widiasih, Dini Hidayat, Hasballah Zakaria, Dody Qori Utama, Maria Komariah, Nenden Nur Asriyani Maryam, Hidayat Arifin, Habsyah Saparidah Agustina, Katherine Nelson

**Affiliations:** 1 Department of Maternity Nursing, Faculty of Nursing, Universitas Padjadjaran, Bandung 45363, Indonesia; 2 General Hospital of Hasan Sadikin, Bandung 40161, Indonesia; odhieny@yahoo.com; 3 Institut Teknologi Bandung, Bandung 40116, Indonesia; fahala@gmail.com (H.Z.); dodyqori@gmail.com (D.Q.U.); 4 Department of Fundamental Nursing, Faculty of Nursing, Universitas Padjadjaran, Bandung 45363, Indonesia; maria.komariah@unpad.ac.id; 5 Department of Pediatric Nursing, Faculty of Nursing, Universitas Padjadjaran, Bandung 45363, Indonesia; nenden.nur@unpad.ac.id; 6 Department of Medical Surgical Nursing, Faculty of Nursing, Universitas Padjadjaran, Bandung 45363, Indonesia; hidayat.arifin@unpad.ac.id; 7 Department of Mental Health Nursing, Faculty of Nursing, Universitas Padjadjaran, Bandung 45363, Indonesia; habsyah.s.agustina@unpad.ac.id; 8 School of Nursing, Midwifery, and Health Practice, Victoria University of Wellington, Wellington 6021, New Zealand; kathy.nelson@vuw.ac.nz

**Keywords:** fetal movement, antenatal care, infant well-being, self-monitoring

## Abstract

Pregnant women are expected to have a high level of awareness when it comes to checking their fetal health and ensuring their welfare. This study explored the experiences of pregnant women in Indonesia who were monitoring their fetal wellbeing during the COVID-19 pandemic. A qualitative descriptive study design with a constructivist paradigm was used. Twenty-two pregnant women were recruited and participated in a semi-structured interview. Analysis of the transcribed interviews used a content, thematic and comparative process. Three themes emerged from the analysis: feelings and responses, changes to the ante natal care service during the COVID-19 pandemic, and the fetal wellbeing monitoring, tools, and methods used. Advice on how pregnant women should conduct fetal wellbeing monitoring during COVID-19 is urgently needed. The results of this study indicate there is a need for interventions to help pregnant women carry out self-fetal wellbeing monitoring in times where they have fewer contacts with health professionals such as during the COVID-19 pandemic.

## 1. Introduction

The 2019 novel coronavirus disease pandemic (COVID-19) has been ongoing for more than two years. Currently, its spread continues worldwide. Based on data from the World Health Organization (WHO), as of 17 September 2021, 226,236,577 people have been confirmed positive for COVID-19 globally [1]. In Indonesia, by September 2021 there had been 4,181,309 people confirmed with COVID-19, and 139,919 deaths [2]. Pregnant women are one of the groups who are susceptible to COVID-19. They are also a group at risk of serious health conditions such as preeclampsia, bleeding, premature rupture of membranes (PROM), and infections that affect the mother and fetus [1]. Similar to many countries, the Indonesian government established rules to prevent the spread of COVID-19 in the form of social restrictions [3]. Most (84%) basic health services could not run optimally, especially at the integrated service posts, and the already limited maternal and neonatal health services were severely restricted. These restrictions impacted the access to and availability of antenatal care (ANC) and classes for pregnant women [3,4]. But within these restrictions, pregnant women still had the right to access ANC services, support, education, and childbirth assistance [5].

Antenatal care delivered by midwives, nurses and doctors is important for pregnant women in order to ensure the health of their unborn babies [6]. In addition to health checks, ANC services provide women with information, education and preparation for childbirth [7]. During the COVID-19 pandemic ANC services underwent several policy changes related to the system, administration, and implementation process. Given that it was known that ANC examinations are important for pregnant women, pregnant women were still expected to adhere to the ANC examination schedule while paying attention to the COVID-19 health protocols [8]. Based on the latest WHO recommendations, pregnant women at low risk should get at least eight ANC checks [9]. Prior to appointments, the pregnant women are encouraged to make prior arrangements with local health workers to reduce the potential for queues during the ANC examinations [10]. Classes for pregnant women can also be offered through adaptations and innovations including telehealth or running the classes online [11]. Pregnant women with good knowledge regarding the condition of their pregnancy can minimize any maternal and neonatal problems [5].

In the COVID-19 pandemic situation, pregnant women are expected to have a high level of awareness to check their pregnancy and ensure the welfare of their unborn babies. Even though there were clinics available, many pregnant women in Indonesia chose not to attend any or few health services, or postponed visits because they were afraid of contracting COVID-19 [10]. Reducing the number of ANC visits increases the health risk for a pregnant woman and the unborn baby [12]. Pregnant women are also expected to self-monitor the health of both themselves and the fetus to reduce the various risks due to pregnancy abnormalities, and to seek assistance if they have concerns. This monitoring is particularly important if women are not attending ANC. The unborn baby in the uterus regularly moves, [13] and part of the monitoring by women is to be familiar with their fetuses’ movement patterns. Known problems experienced by pregnant women during the pandemic include anxiety due to being pregnant in the midst of a pandemic, unpreparedness for childbirth, the threat of the pandemic itself, and the impact of COVID-19 [3]. To date, little is known about how pregnant women in Indonesia are monitoring the welfare of their health and that of their unborn babies. This study was designed to explore the experiences of pregnant women regarding ANC and monitoring fetal wellbeing during the COVID-19 pandemic.

## 2. Materials and Methods

### 2.1. Design

The study design used a qualitative descriptive approach with a constructivist paradigm. This approach seeks to describe all situations and circumstances as they are, including what is still happening or being carried out at the time of the study [14]. The constructivist paradigm involves the active role of the social and practitioner values in shaping the descriptions and statements expressed by the participants [15]. The research team are health professionals with expertise in maternity nursing, gynecology and obstetrics, and biomedical engineering, with an interest in childbirth. The research team consisted of men and women. No team member had a relationship with any participants in the study.

### 2.2. Participant and Setting

Purposive sampling was used to recruit the women. The participants were selected from the Public Health Center (PHC) because the pregnant women who registered in the PHC were known to have a lower risk of pregnancy problems and a lower risk of exposure to COVID-19. Village midwives assisted with recruiting participants. The inclusion criteria were women in their third trimester of pregnancy, being in good health, having no history of a high-risk pregnancy, and attending ANC regularly. All women who were approached consented to participate in the study. The participants were from an area of the Public Health Center, Sumedang Regency, West Java Province, Indonesia.

### 2.3. Ethical Considerations

This study received ethical approval from the Health Commission Ethics Committee of Universitas Padjadjaran, Indonesia (Ref 1041/UN6.KEP/EC/2020). Participants were required to give their written consent free of coercion to participate. They could withdraw from the study without giving reason, and with no impact on their health care, and could decline to answer any of the questions. Furthermore, the researchers maintained the participants’ privacy throughout the interview process. All of the interview data was de-identified at the time of transcribing, with participants being named according to number such as P1, P2 and so forth. The study did not have the potential to harm the participants physically or mentally.

### 2.4. Data Collection

A semi-structured interview schedule was developed by three experts (a medical specialist in obstetrics and gynaecology, a lecturer of nursing in maternity, and a midwife who worked at a public health centre). The draft schedule was pre-tested with three pregnant women. Following the pretest, minor changes to wording of some questions was made to make the questions clearer. The pretest provided an opportunity for the interviewers to become familiar with the schedule. The interviews were conducted between November 2020 to January 2021 by four researchers. The researchers paid attention to the principles of social distancing when the face-to-face interviews took place. The interview began by building trust between the participants and interviewers. This involved introductions, gaining consent and obtaining permission to audio-record the interview. The interviews then proceeded with questions covering the pregnant woman’s experience when engaged in ANC and how and when they undertook fetal welfare checks during the COVID-19 pandemic. Each participant was interviewed for approximately 20 min. In addition to recording the sessions, the interviewers took notes of their observations.

At the end of each interview, the interviewee repeated what the woman had said to confirm they had accurately understood what the woman had shared. No women made any changes at this point. Triangulation of the interview and field work observation data was undertaken to enrich the data [16]. Recruiting participants ceased when the data reached saturation.

### 2.5. Data Analysis

The researchers transcribed the results of the interviews into Microsoft Word. Following the transcription, the researchers then coded and analyzed the data using the Comparative Analysis for Interview (CAI) approach [17]. The CAI approach was developed based on two qualitative analysis methods, namely the Qualitative Analysis Guide of Leuven [18] and the Comparative Analysis for Interview [19]. This approach includes four steps: precoding, coding, formulating the themes, and presenting the themes. The first step included reading the 22 transcriptions at least five times, selecting meaningful sentences from the transcriptions (for example, “the experience of antenatal examination during a pandemic”), writing a narrative interview summary based on the researcher’ s views, reviewing the narrative text, and matching the narrative with the questions. The second step in the analysis was coding the meaningful sentences using NVivo version 12 software, grouping the same data into sub-categories, and developing the categories. The third step was to formulate the themes based on the categories. The researchers’ weekly regular meeting was conducted to discuss the findings. The final step was to present the themes with quotes from the participants. To enhance the quality and transparency of the study results and the associated reporting, the researchers applied the Standards for Reporting Qualitative Research (SRQR) [20].

## 3. Results

Twenty-two women participated in the study. The average age of the participants was 27 years (range 19–40). Most (n = 16) participants had a primary level of education, four had secondary school education and three university or high education. Most (n = 17) of the participants were housewives, three worked in private companies, and two were midwives. Their pregnancy data indicates that for 12 participants it was their first pregnancy, for five their second, and for five it was their third or more pregnancy. For one participant it was her 8th pregnancy. Of those who had been pregnant previously, two had had a miscarriage. The participants gestational age at time of interview ranged from 28–37 weeks). Table 1 details the data for each participant.

Three themes emerged from the analysis related to the experiences of pregnant women when conducting ANC visits and examining fetal welfare during the COVID-19 pandemic. The themes were (1) feeling and responses, (2) changes to the ANC service during the COVID-19 pandemic, and (3) fetal wellbeing monitoring, tools and methods (Table 2). The following is a description of each theme and the relevant signposted participant quotes.

### 3.1. Feelings and Responses

The analysis revealed that the participants expressed mixed feelings about their pregnancy during the pandemic. Most participants experienced difficulties with feelings of worry, fear, confusion, boredom, and anticipation. Some of the participants felt happy and grateful for their pregnancy during the pandemic. Three sub-themes that support the theme of feelings and responses emerged: (1) positive feelings, (2) negative feelings, and (3) their response to COVID-19.

#### 3.1.1. Positive Feelings

The positive feelings expressed by the participants included feeling happy because during the period of social distancing and territorial restrictions, some participants got pregnant after waiting for years. In addition, many participants expressed that they were grateful for being able to rest at home during their pregnancy (Quotes 1–2).

#### 3.1.2. Negative Feelings

The negative feelings expressed by all participants included a fear of contracting COVID-19, anxiety, fear of childbirth, confusion when trying to find a safe delivery place, and being bored due to the COVID-19 pandemic (Quotes 3–5).

#### 3.1.3. Response to COVID-19 Prevention

In addition to expressing their feelings, participants shared particular responses in light of the COVID-19 pandemic. Several shared that they have become fastidious about health practices, including routine hand washing, wearing masks, avoiding crowds, maintaining distance, and not traveling (staying at home only). The participants also said they were more vigilant if they experienced health problems such as the flu or a cough because they feared that the symptoms were the symptoms of COVID-19. The participants said that during pregnancy in the middle of the ongoing pandemic, things were stricter regarding maintaining health and cleanliness (Quotes 6–7). One participant revealed that she maintained her health by eating nutritious foods such as vegetables and other healthy foods.

### 3.2. Changes to the ANC Service during the COVID-19 Pandemic

The participants shared a variety of experiences to do with the changes to the ANC service during the COVID-19 pandemic. The analysis determined there to be three sub-themes: (1) wearing personal protective equipment, (2) queues and restrictions on the number of patients, and (3) the integrated service post providing active services during COVID-19.

#### 3.2.1. Wearing Personal Protective Equipment

The participants reiterated that the use of personal protective equipment is a must in the COVID-19 pandemic. They expressed the importance of using PPE such as masks and hand sanitizers, in addition to maintaining cleanliness by regularly washing their hands during the ANC examinations (Quotes 8–10).

#### 3.2.2. Queues and Restrictions on the Number of Patients

The participants revealed that during the COVID-19 pandemic, services were open for shorter periods and there were restrictions on the number of patients who could have ANC examinations on any one day. The cap on the number of women who could be seen at a clinic meant participants had to come early to ensure they could get an examination (Quotes 11–14). The participants who were familiar with internet technology felt that the online service helped them to manage their time effectively.

#### 3.2.3. The Integrated Service Post Provides Active Services during COVID-19

The participants noted that the services available at the integrated service post near their houses had become more active. During the COVID-19 pandemic, integrated post services were activated in order to conduct examinations for pregnant women and children. At these integrated service posts, midwives carry out ANC checks for pregnant women and provide medicines and vitamins. The participants felt that having an integrated service post made things easier, as it could mean they did not have to go to the PHC, where there were long queues (Quotes 15–16).

### 3.3. Fetal Wellbeing Monitoring, Tools and Methods

The participants shared multiple perspectives and experiences related to fetal well-being and monitoring. These experiences clustered around three sub-themes: (1) the motivation to monitor the fetal welfare, (2) the knowledge of fetal monitoring methods, and (3) the tools used for fetal monitoring.

#### 3.3.1. Motivation for Monitoring Fetal Wellbeing

The participants expressed a variety of reasons and motivations related to monitoring fetal wellbeing. Most of them stated that they were curious about the pregnancy process, especially women who were pregnant for the first time. They wanted to know the fetus’ s condition in the womb because the fetus was not visible to the naked eye, and the participants could not always feel the active movements of the fetus. Another thing that motivated them to monitor things was the desire to know the fetus’ s health, growth and development. They felt happy if they could find out their condition (Quotes 17–19). On the other hand, knowing the fetus’ s condition made some participants’ anxious because they knew that the position of the fetus was not normal. Some participants expressed that self-monitoring was a burden. These participants shared that they did not engage in fetal monitoring because they were busy working, had no money, and did not know if the PHC had open services during the COVID-19 pandemic (Quote 19).

#### 3.3.2. Knowledge of Fetal Monitoring Methods

Many participants knew that fetal monitoring is carried out with special tools such as an ultrasound. The participants who had an educational background in the health area, such as the midwives, knew how to do independent fetal monitoring. This monitoring involved regularly checking fetal movements manually at home. These participants said that they monitored the fetus when they woke up, or at certain hours scheduled between 8:00 a.m. and 11:00 a.m. Some participants also performed stimulation if they did not initially feel any fetal movement. (Quotes 20–22).

#### 3.3.3. The Tools for Fetal Monitoring

The participants revealed that they conducted fetal monitoring at home using manual methods; for example, they manually counted and felt the unborn baby’s movements for about two hours. Another method they used involved a rubber band to track the movements. Women moved the rubber band from right to left hand when they felt any fetal movements. In the current era of technological advances, most participants expressed hope that in the future that sophisticated fetal monitoring devices that are light and small and easy to operate independently would be available. They considered that such devices would result in there being no need to go to the health services (Quotes 23–24).

## 4. Discussion

In this study, three themes were found that provide information related to the experiences of pregnant women in Indonesia about ANC and the monitoring of fetal wellbeing during the COVID-19 pandemic. The themes reported by the pregnant women include feelings and responses, changes to the ANC service during the COVID-19 pandemic, and fetal wellbeing monitoring, tools and methods.

The feelings and responses expressed by the participants in this study are the concerns of pregnant women for their own health and that of their unborn child. These findings are in line with the longitudinal survey conducted by Naurin et al. (2019). They examined approximately 4300 pregnant women in Sweden who reported that pregnant women experience dramatically increased concerns for their own health and that of their babies [21]. A similar finding was reported by Ng et al. (2020) who conducted a cross-sectional survey of 324 pregnant women at an antenatal clinic in Singapore. These authors argued that there was a lack of accurate information about the impact of COVID-19 on pregnancy. Their results indicated there were increased levels of depression, anxiety, and stress in pregnant women [22]. While our study did not capture findings regarding depression, it is apparent from the findings that women’s mental health is impacted, with many expressing increased anxiety and raised stress levels. The availability of information about the psychological condition of pregnant women during the COVID-19 pandemic and the need for high-tech self-fetal monitoring methods are important findings for healthcare in Indonesia.

In contrast to this negative impact of COVID-19, an important finding is that the pandemic also gave rise to some participants feeling positive about being pregnant at this time. These participants were happy with the government policy related to social distancing. In addition to the efforts undertaken to prevent the transmission of COVID-19 carried out by pregnant women, all of the participants complied with and carried out the health protocols recommended by the government, such as wearing masks, washing their hands frequently with soap, carrying hand sanitizer while traveling, and avoiding crowds. This was felt to be both a habit and a new thing that was rarely done before the pandemic. Pregnant mothers had come to realize the importance of prevention because the disease would affect both themselves and their babies.

In this study, we found that participants experienced of changes of the ANC services during the COVID-19 pandemic. These changes included the new habit of using PPE, queues and limits on the number of patients, changes to the service hours, and the services at integrated service posts around their home become more active. A previous Indonesian-based study showed that pregnant women changed their behavior at ANC and actively engaged in using masks, hand sanitizers, and washing their hands [23]. In addition, the health services in the community have become more active in terms of conducting ANC examinations including home visits and a community clinic [24]. Other known changes included shorter ANC service hours. To avoid queues at the health services, pregnant women are expected to arrive on time (not too early) and according to a predetermined schedule [24]. However, facilities related to booking within the ANC service hours are still limited to several urban areas and have not reached rural areas. In this study, participants revealed that queues of pregnant women waiting for ANC examinations were still occurring. A need for the use of technology to facilitate making appointments without having to queue for this access is evident. During the ongoing COVID-19 pandemic, an intervention with a technological approach is needed to make it easier for pregnant women to carry out ANC.

Knowing the unborn baby’s health status is the biggest reason why pregnant women monitor their fetal wellbeing in terms of heart rate and fetal movement. Pregnant women know about self-fetal monitoring, especially those who work as health workers. They monitor themselves manually and use tools. The ability of self-fetal monitoring is proven to reduce the risk of fetal death. This was proven in a study in Liberia focusing on 470 pregnant women who were asked to carry out the independent monitoring of their unborn baby using a sonic aid. The pregnant women were trained because of the lack of health workers in the country [25]. In Indonesia, more than 80% of deliveries are carried out at the health services [26], so there are no problems like there are in Liberia. However, independent fetal monitoring is still needed because of the high number of risks due to pregnancy [27] and the high rate of intra-uterine fetal death (IUFD) in Indonesia [28]. Fetal welfare monitoring aims to increase the maternal awareness of fetal health so that it is monitored regularly.

The participants’ expectations regarding the methods for monitoring the fetus’ s condition focused on the use of the latest technology that was easy to apply, simple, and familiar, like cell phones. The results indicated that the participants need a fetal monitoring tool that can be used independently. The expectations of the participants in this study are in line with a previous cross-sectional study conducted to examine women’ s attitudes regarding self-fetal monitoring using non-invasive electronic devices [29]. The study was conducted on pregnant women in Germany who had previously participated in a telemedicine program. The pregnant women revealed the need for electronic devices for fetal monitoring independently, while the results were additionally monitored by their doctors. The results of the two studies mutually reinforce and illustrate that technology, especially wireless and internet devices, is a need globally, not only in developed countries such as Germany but in all countries as well. Technology should be involved in various aspects of life, including health. Self-fetal monitoring with a technological approach will improve the monitoring of fetal well-being overall and the accuracy of the monitoring results compared to manual observation methods. Abnormal conditions in terms of the heart rate and fetal movement will be detected immediately, reducing the risk of fetal death in the womb.

### 4.1. Strengths and Limitations

This study has been conducted rigorously. Audit trails were maintained during data analysis and the use of multiple quotes to demonstrate themes demonstrates the credibility of the findings. The inclusion criteria and recruitment process may have meant that the sample was skewed towards women who were actively engaged in monitoring their and their unborn child’s health. Obtaining a sample any other way was not possible because of COVID-19. Despite this limitation, the findings reveal important insights into the difficulties and delights of being pregnant in Indonesia during a pandemic.

### 4.2. Implications for Practice

The COVID-19 pandemic has had a massive impact on all aspects of life, including pregnant women having to monitor their pregnancy more independently. There is a need for increased instruction and clear guidance on how to self monitor and when to seek help when concerned about fetal movement and activity. This situation increases the need for women to have access to technology that they can use to self-monitor their pregnancy. Such technology is not readily available in Indonesia. This study provides an insight into the need for researchers to develop fetal well-being monitoring technology that is affordable, easy to use at home, and that provides accurate information. Such technology would help pregnant women self-monitor, while also making efforts to prevent pathogen transmission to both the mothers and fetuses. In addition to finding ways of supporting home-based monitoring of the fetus, maternity providers need to be cognizant of the mental health needs of pregnant women. Health workers should also consider using social media and forming networks for pregnant women to share experiences related to the pregnancy process.

## 5. Conclusions

During the COVID-19 pandemic, pregnancy anxiety and changes to the ANC services in Indonesia have posed challenges for pregnant women. These challenges resulted in pregnant women having a greater role in monitoring their pregnancy using self-fetal monitoring techniques. Clear guidelines and standardized practice are needed regarding self-monitoring. Women need fetal self-monitoring tools that are easy to use, accurate, and technologically familiar. The results of this study provide important information for health workers, as they provide evidence of the vulnerability of women being pregnant during a pandemic. Not only are women and their fetuses vulnerable to the risk of COVID-19, but women also experience additional psychological distress. The development of self-fetal monitoring tools should be prioritized by researchers and academics in collaboration with industry. Further research is needed into how women in different health settings monitor their pregnancy during COVID-19 and how they can maximise their health and well being.

## Figures and Tables

**Table 1:** Demography Characteristic (n = 22).

ID	Age	GPA	Gestational Age	Education Level	Occupation
P1	33 Years old	G2P1A0	28 weeks	High Education	Housewife
P2	31 Years old	G3P2A0	32 weeks	Secondary Education	Housewife
P3	36 Years old	G1P0A0	28 weeks	Primary Education	Housewife
P4	25 Years old	G1P0A0	28 weeks	High Education	Midwife
P5	22 Years old	G1P0A0	33 weeks	Primary Education	Housewife
P6	40 Years old	G3P2A0	36 weeks	Primary Education	Housewife
P7	39 Years old	G8P6A1	36 weeks	Primary Education	Housewife
P8	19 Years old	G1P0A0	29 weeks	Primary Education	Housewife
P9	20 Years old	G1P0A0	36 weeks	Primary Education	Housewife
P10	22 Years old	G2P1A0	37 weeks	Primary Education	Housewife
P11	19 Years old	G2P1A0	34 weeks	Primary Education	Housewife
P12	21 Years old	G1P0A0	28 weeks	Primary Education	Midwife
P13	26 Years old	G2P1A1	37 weeks	Secondary Education	Private
P14	21 Years old	G1P0A0	28 weeks	Primary Education	Private
P15	20 Years old	G1P0A0	28 weeks	Primary Education	Housewife
P16	27 Years old	G2P1A0	39 weeks	Primary Education	Housewife
P17	22 Years old	G1P0A0	28 weeks	Primary Education	Housewife
P18	30 Years old	G3P2A0	30 weeks	Primary Education	Private
P19	22 Years old	G1P0A0	29 weeks	Secondary Education	Housewife
P20	24 Years old	G1P0A0	29 weeks	High Education	Housewife
P21	40 Years old	G4P3A0	28 weeks	Primary Education	Housewife
P22	34 Years old	G1P0A0	36 weeks	Primary Education	Housewife

P: Participant; G: Gravidarum; P: Partus (delivery); A: Abortus (a miscarriage).

**Table 2:** Distribution of themes and sub-themes (n = 22).

Theme	Sub-Theme	Quotes
Feeling and Responses	Positive feelings	Q1: “During the COVID-19 pandemic, I felt happy… because I could get pregnant… I have been waiting for this pregnancy for seven years… thank God… I am currently pregnant on my first child …” (P1).
		Q2: “I am grateful… because during COVID-19, I only went to the office once a week… I couldn’ t afford to go to the office continuously…” (P13).
	Negative feelings	Q3: “I’ m scared and worried mom… I’ m afraid of getting infected. She said, pregnant women are very vulnerable to contracting COVID-19. I was worried and scared” (P19).
		Q4: “I’ m bored… because my activities during COVID-19 are limited… I can’ t go anywhere. In addition, during COVID-19, my work stopped completely” (P14).
		Q5: “The difficulty during COVID-19 can’ t go anywhere. During the restriction program, it is not permitted to ride a motorized vehicle in couple. Activity becomes limited and becomes difficult” (P17).
	Response to COVID-19 prevention	Q6: “I am quite worried about this condition. I am also currently pregnant and in the range of contracting COVID-19. So every time I go, I have to wear a mask, I always carry hand sanitizer... every time I get home... I definitely take a shower... but I mostly stay at home... I’ m afraid if I catch COVID-19 out there…” (P20).
		Q7: “… Even though… I get scared when I cough and run cold. I immediately eat a lot of food... which can increase body immunity... besides that, maintaining cleanliness and maintaining distance are also very important...” (P18).
Changes to the ANC Services during the COVID-19 Pandemic	Wearing Personal Protective Equipment (PPE)	Q8: “Now, if I want to go outside, I have to use a mask, wash my hands diligently. Thus, I could not get infected by COVID-19 and maintain the health of the fetus...” (P10).
		Q9: “Yes, if I want to check (ANC), I have to wear a mask… I have to wash my hands and use a hand-sanitizer...” (P15).
		Q10: “To prevent the transmission by wearing a mask. Then, limit the activity of going out of the home …” (P4).
	Queues and restrictions based on the number of patients	Q11: “The services now feel more complicated during COVID-19. If I go to the hospital, I have to queue and there was a quota... now it feels more complicated and difficult... In the past, it was enough to come and take the queue. There was no quota of restrictions…” (P1).
		Q12: “The barrier is the limitation on the number of patients. Before COVID-19, it wasn’ t. Now services are limited to 15 people per day. Thus, I have to go early …” (P17).
		Q13: “At the Public Health Center (PHC), the queues are always long. I went at 8 am and arrived at home at 1 pm. Moreover, the quota is limited, if I went late, I could not be checked because the quota has run out…” (P6).
	Changes to the service hours	Q14: “Service is now faster and close earlier… It was not like before… I have to come early,…” (P9).
	The integrated service post provides active services during the COVID-19 pandemic	Q15: “In every area there is an Integrated Service Post (ISP)... Midwives carry out checks at the ISP, provide medicines, and consultations can be done there... so now, there was no need to go to the PHC” (P16).
		Q16: “Services at ISP are now more active. I can check (ANC) at the ISP. In the past, examinations were only for children …” (P8).
Fetal Wellbeing Monitoring, Methods, and Tools	Motivation for monitoring fetal wellbeing	Q17: “I want to know if my unborn baby is healthy in the womb. I always keep my food and activities. I want to take care of my unborn baby and healthy…” (P2).
		Q18: “I want my unborn baby healthy, and to have active movements during pregnancy. From the ultrasound results, doctor said that my son is a boy…” (P8).
		Q19: “I want to know the condition of my unborn baby... because from the results of the previous control, the position of the fetus in the womb is not normal…” (P6).
	Knowledge of fetal monitoring	Q20: “…counting fetal movements regularly... if it moves, alhamdulillah (An expression of gratitude). It means that my unborn baby is healthy in the womb. I usually check it every morning when I wake up and finished my breakfast. I definitely check it every time I wake up in the morning…” (P22).
		Q21: “I felt the movement… I usually touch my stomach to make sure it moves or not… I am afraid that there will be no movement in my unborn baby …” (P19).
		Q22: “a better examination using ultrasound... Fetal movement and development can be seen from ultrasound …” (P17).
	Methods and tools for fetal monitoring	Q23: “When I’ m at home, I’ m used to counting fetal movements using a rubber band. When the fetus moves, I move the rubber band on another hand …” (P13).
		Q24: “My hope is like that, it’ s not too difficult… It can be monitored with the equipment without having to go to a midwife. Now is the era of technology... I want something like a handphone, which is simple and not heavy…” (P6).

Q: Participant’s quote. P: Participant.

## Data Availability

The data presented in this study are available on request from the corresponding author.
